# Artificial intelligence driven multi-omics framework identifies COL6A3 as a diagnostic biomarker and a putative gene target modulated by Embelin in Colorectal cancer

**DOI:** 10.3389/fonc.2026.1711079

**Published:** 2026-02-02

**Authors:** Prashanth S Javali, Kavitha Thirumurugan

**Affiliations:** Structural Biology Lab, Pearl Research Park, School of Biosciences & Technology, Vellore Institute of Technology, Vellore, Tamil Nadu, India

**Keywords:** apoptosis, artificial intelligence, COL6A3, colorectal cancer, diagnostic biomarker, embelin, machine learning, multi-omics

## Abstract

**Background:**

The third leading cause of death worldwide is colorectal cancer due to a lack of early detection biomarkers and therapeutic small molecules. Advances in systems biology offer a combination of multi-omics and Artificial intelligence to discover the potential biomarkers and targets.

**Methods:**

We used a combination of *in silico* and *in vitro* methodologies to identify potential biomarkers and a putative mediator of Embelin in colon cancer treatment. The human colorectal cancer (gene expression profiling by array) datasets were analyzed by using Weighted Gene Co-expression Analysis (WGCNA), and predictive AI models were trained by three algorithms (LASSO, SVM-RFE, RF). All three algorithms predicted COL6A3 as a common hub gene. qRT-PCR was used to analyze the expression level of COL6A3 along with apoptosis markers in HCT116 cell lines (human colorectal cancer) by treating Embelin in a dose-dependent manner.

**Results:**

Trained model predicted COL6A3 as a prominent hub gene across all three ML algorithms with high cross validation accuracy (AUC values: > ~0.90), showing the accuracy of predictions and feature selections of the trained model. Embelin treatment results in the upregulation of pro-apoptotic markers (BAX, CASPASE3) and the downregulation of anti-apoptotic genes (BCL2, PI3KCA). These findings suggest that COL6A3 is a candidate biomarker and a potential mediator of embelin activity.

**Conclusion:**

This study underscores the integration of AI, multi-omics, and *in vitro* studies for the discovery of candidate biomarkers and mechanistic insights into pathway modulation by Embelin in colorectal cancer. The research successfully identified and validated the role of COL6A3 as a potential biomarker and putative target modulated by Embelin in colon cancer.

## Graphical Abstract

**Figure:**
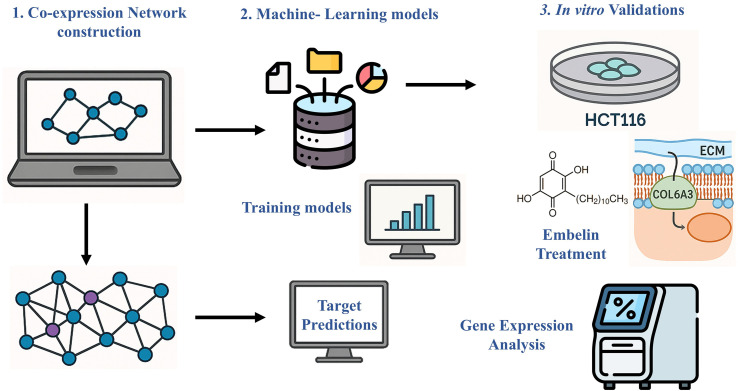

## 1 Introduction

The most common third leading cause of cancer-related deaths worldwide is colorectal cancer (CRC), which originates as adenomatous lesions in colon tissues and develops into tumours. It is a type of cancer with the capacity to spread to vital organs such as liver, lungs and some parts of the digestive system (1). By the end of 2040, an estimated 3.2 million cases of CRC are expected worldwide, particularly in China and the United States, indicating a high number of cases (2). Key risk factors for CRC include personal and family history, genetic mutations (APC gene) and chronic inflammation (3). Additionally, Molecular Pathophysiology and Epidemiology (MPE) explores how genetics, microbiome, diet, lifestyle, and disease progression interact in CRC development (4). Most cancers arise from dysfunctions in numerous gene products rather than inherited mutations alone (5). Besides inherited mutations, many cancers result from genetic alterations in BRAF, KRAS, PI3KCA, growth factors (EGF, VEGF, IGF-1) (6, 7), growth factor receptors, kinases (8), cytokines (TNF, IL-1, IL-6) (9), and other transcription factors such as NF-kB, APC1, STAT3 and PARP (10). Among these, NF-κB-driven inflammation plays a central role in CRC, as it is triggered by diet, stress and environmental pollutants. Early diagnosis of colorectal cancer is typically performed using tissue biopsy and colonoscopy techniques, with treatment strategies depending on the stage of the cancer. Besides surgical intervention, chemotherapy and targeted immunotherapy are prominent; recent studies show that aspirin, a common nonsteroidal anti-inflammatory drug, can prevent colon cancer progression in PTGS2-positive and PI3KCA-mutated CRC patients (11, 12). Surgery and chemotherapy are standard treatments, with chemotherapeutic agents causing DNA damage or activating various signaling pathways, such as those controlling the cell cycle, translation, and DNA repair (13). The effectiveness of cancer drugs for colorectal cancer (CRC) patients varies depending on the cancer subtype, as demonstrated by multiple studies, including those on MPE (14). Chemotherapy has several adverse effects that impact quality of life due to increased cytotoxicity and drug resistance. The high cost of chemotherapeutic drugs prompts researchers to seek alternative, cost-effective compounds with lower risks (15, 16). Consequently, natural compounds are gaining research interest due to their pharmacological properties and biological effects, which can mitigate the adverse effects of chemotherapeutics (17). Traditionally, many herbal formulations have demonstrated anticancer properties against various cancers, including CRC, with most natural compounds being derived from plants and marine sources (18, 19). Their unique chemical structures make them potent anticancer compounds capable of modulating the cancer-causing signaling pathways (20). Most phytocompounds exhibiting anticancer properties are phenolic compounds, often working synergistically with other constituents (21). Although many phytocompounds are inherently anticancer, further research is necessary to understand the mechanisms and pathways through which they can modulate and prevent cancer progression.

One such natural compound is embelin, a false black pepper derivative (*Embelia ribes*) extracted from various parts of the plant. As a benzoquinone derivative, it exhibits many pharmacological properties, such as anti-inflammatory, anti-tumour, antioxidant and others, which are all documented in ancient texts by Ayurvedic physician Sushrutha (22). Notably, embelin is identified as X- linked inhibitor of apoptosis protein (XIAP) (23) and is capable of inducing autophagy and apoptosis in various cancer cell types (24). Additionally, embelin can modulate most protein kinases, oncogenic transcription factors, and cytokines (25). Studies have shown that when combined with radiation therapy, embelin can enhance tumour suppression. It has been demonstrated that embelin regulates the extrinsic apoptotic pathway by inhibiting TNF-α, TNF receptor-1, and TRADD2, thereby reducing TNF-α levels in breast cancer cells. Moreover, TRAIL sensitivity is restored in resistant cancer cells by inhibiting XIAP in pancreatic, nasopharyngeal, and inflammatory breast cancers (26). Furthermore, embelin enhances TRAIL-mediated apoptosis by downregulating FLIP in glioma cells, as well as survivin, Bcl-2, and FLIP in lung cancer cells A549. In leukaemia, it promotes TRAIL-induced apoptosis by upregulating DR4 and DR5 (27). Embelin also mediates the intrinsic pathway, inducing apoptosis via the mitochondria-dependent pathway in various cancer cells (28).

**Figure 1:**
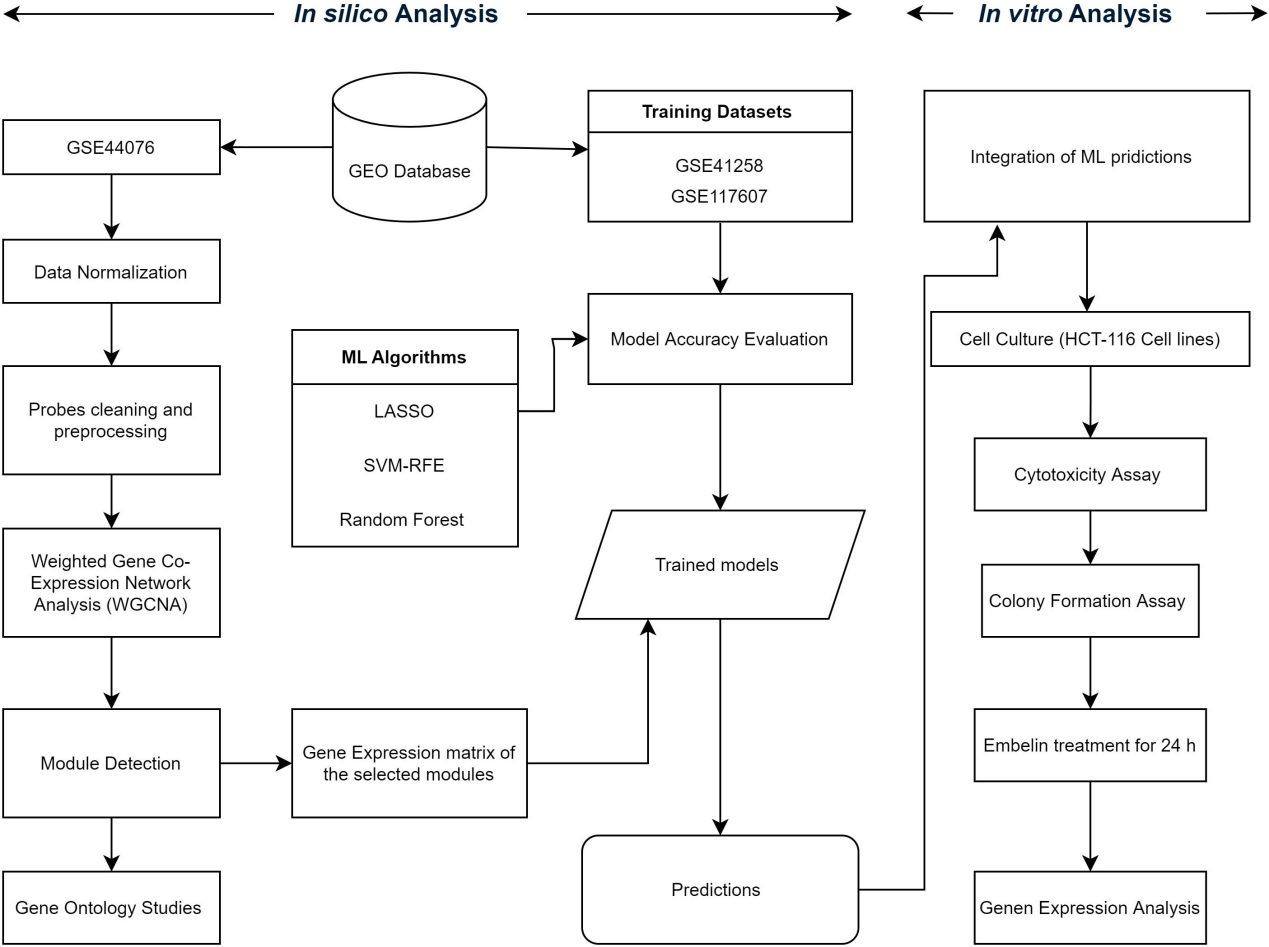
Integrated approach to discover the therapeutic target of embelin in colon cancer.

Recent advancements in systems biology open a new lane for the discovery of potential biomarkers and therapeutic targets for disease diagnosis and prognosis, including cancer. The availability of microarray data enables progress in biomarker discovery. Systems biology provides various techniques to analyse the transcriptomics datasets efficiently: weighted gene co-expression network analysis (WGCNA) finds the co-expression pattern of multiple genes in different conditions to identify the biomarker and potential therapeutic targets for the particular disease. In this study, we aimed to identify genes that are involved in the development and progression of colon cancer and to assess their potential as therapeutic targets and diagnostic biomarkers. To achieve this, we employed a comprehensive integrative approach, a combination of *in silico* and *in vitro*. Initially, publicly accessible microarray datasets were analyzed using WGCNA to construct gene co-expression networks and identify modules significantly associated with the CRC phenotype. Subsequently, machine learning algorithms were utilized to predict prominent hub genes by training predictive models on training datasets capable of distinguishing between healthy and tumour states with high accuracy. From these candidates, a key gene was selected for further validation from the validation dataset. From our previous study, we identified that embelin targets the PI3K and AKT pathway to treat Ulcerative colitis (29). Thus, the therapeutic relevance of embelin in colon cancer was investigated through *in vitro* functional assays. Functional assays, including MTT and colony formation assays, were performed to assess the cytotoxic and anti-proliferative effects of embelin in colon cancer cells. Additionally, gene expression analysis was carried out using quantitative reverse transcription polymerase chain reaction (qRT-PCR) to confirm the molecular modulation of the target gene. Overall workflow is illustrated in **Figure 1**. Collectively, this study aimed to identify the key biomarker of the embelin-mediated pathway in CRC with experimental support.

## 2 Materials and methods

### 2.1 Data acquisition and preprocessing

The microarray dataset (GSE44076), part of the COLONOMICS project, includes a total of 246 samples (148 Healthy and 98 Tumour) and is based on the Affymetrix Human Genome U219 Array platform (GPL13667) was obtained from the GEO database (30). We utilized R Studio with R version 4.5.2 and the WGCNA Bioconductor package for the analysis. Initially, we applied the RMA normalization method to correct the background and normalize the data, using the hgu219.db annotation file to annotate the probes of the gene expression matrix. Following this preprocessing, we transformed the expression matrix using a logarithmic base of two to achieve a more normal distribution and ensure consistent variation. This normalized expression matrix will serve as the input for WGCNA. We used various packages of R, such as limma v3.54.0, GEOquery v2.66.0 and ggplot2.

### 2.2 Uncovering biologically significant gene modules through WGCNA

To identify physiologically significant gene modules associated with colon cancer, Weighted Gene Co-expression Network Analysis was performed using the WGCNA package in R on normalized, log_2_-transformed expression data from the GSE44076 dataset. WGCNA is a comprehensive tool for performing weighted and unweighted correlation network studies on complicated datasets, facilitating gene screening, module structure analysis, and the assessment of gene-module interactions (31). The top 5,000 genes with the most variability were chosen using the median absolute deviation (MAD) to reduce noise as a standard practice in WGCNA to ensure a co-expression network is built only upon the genes with significant biological relevance, and reduces the noise and smoothens the calculation of the pairwise Pearson correlation matrix for the entire gene expression matrix. Since we did not find any outlier samples, we included all the samples for further analysis after examination for outliers. These correlations were transformed into an adjacency matrix using a soft-thresholding power (β), which emphasizes strong correlations while maintaining network scale-free topology, a key principle in biological network analysis (32). To identify the soft-threshold (β) value to produce scale-free topology (fit index >0.85), we utilized the pickSoftThreshold function of the WGCNA package. Later, the interconnectivity of the network is analysed by transforming the adjacency matrix into the Topological Overlap Matrix (TOM). A minimum of 30 gene groups were identified based on hierarchical clustering on TOM dissimilarity by using the dynamic tree cut method of the WGCNA package. The modules that have similar expression patterns are correlated and combined with a cutoff greater than 0.75 by using the Eigengene correlation method. Later, all module eigengenes were linked to their respective clinical features and the modules that were strongly related to colon cancer were chosen for further analysis.

### 2.3 Uncovering biological functions of co-expression modules

We utilized web-based platform sources such as DAVID (33) and Enrichr (34) by setting p – value<0.05, adjusted with the Benjamini-Hochberg method to conduct functional gene ontology studies for the prominent genes identified from the WGCNA modules. The genes that fall under these modules exhibit their majority role in cancer-causing pathways and biological processes such as extracellular matrix organisation, collagen metabolic processes, cellular metastasis, and angiogenesis.

### 2.4 Identification of key diagnostic genes through multi-algorithm machine learning approaches

Three different algorithms were used in a comprehensive machine learning methodology to find diagnostic biomarkers that can distinguish between normal tissues and colon cancer tissues: Random Forest (RF), Support Vector Machine-Recursive Feature Elimination (SVM-RFE), and Least Absolute Shrinkage and Selection Operator (LASSO). The predictive AI models were trained by utilizing the two different training datasets obtained from the GEO database (GSE41258 and GSE39582). Initially, these two datasets were normalized and log2 transformed to reduce noise and maintain consistency (35–37). Since these two datasets were from different platforms, we performed the cross-platform normalization by keeping the common gene probe sets between the two arrays in order to harmonize the two datasets for further analysis. Later, we utilized the distinct annotation files of the respective platforms (hgu133a.db and hgu133plus2.db) in order to map the common probe identifiers with the gene symbols or Entrez IDs. The median expression value of each gene was calculated to avoid mapping of multiple probe sets to the same gene symbol. Later on, the harmonised expression matrix from two platforms was merged into a single expression matrix after removing batch effects using the ComBat package. This expression matrix was used as a training dataset to train the predictive AI models. To ensure the equal class distribution of tumour and normal samples between the training and testing sets, we leveraged a stratified train-test split (70:30) to avoid the influence of any single dataset on the trained model. Since one of the training datasets (GSE39582) has an imbalance in sample types, we ensured class-weighing during training the model, which avoids the bias towards the majority class by assigning a higher penalty for misclassification.

In this study, LASSO (Least Absolute Shrinkage and Selection Operator) was chosen due to its effectiveness in high-dimensional settings, which uses an L1 penalty to choose features efficiently by assigning zero coefficients to less significant genes, which avoids the overfitting (38, 39). As far as biological datasets were concerned, the minor modulations in the expression levels of genes will affect the diagnostic implications, ranking the genes based on their gene expression levels and feature discriminative potential is very important (41). So we utilized the Support Vector Machine- Recursive Feature Elimination (SVM-RFE) algorithm, which can remove the features with lower weights and their influence on classification (40). Other than LASSO and SVM-RFE, we utilized Random Forest algorithm, which provides robustness against overfitting by measuring the feature importance based on mean decrease in Gini impurity (42). The stability of all three algorithms was evaluated with ten-fold cross validation, and the constant gene identified from all three algorithms was considered as a reliable biomarker, which avoids the bias of influence of one single method of selection (43). Various Python packages were utilized to train the models and obtain the predictions from the trained AI models, such as LassoCV, Pandas (v1.5.3), numpy (v1.23.5), xgboost (v1.7.4) and Scikit-learn (v1.2.2) and the visualization is done by using matplotlib (v3.7.1) and seaborn (v0.12.2). After training the predictive AI models, we subjected the gene expression matrix of cyan and purple modules to predict the prominent gene and combined all the predictions from each algorithm. Identification of overlapping genes between all three was considered as a hub gene, and this target gene was further utilized to understand its role in the pathogenesis of colorectal cancer and how embelin can modulate the expression level of this particular gene in preventing cancer progression.

### 2.5 *In vitro* validation of embelin in HCT116 colon cancer cells

#### 2.5.1 Cell line and culture conditions

We utilized HCT116 human colorectal cancer cell lines for an *in vitro* study to validate the *in silico* predictions, and we cultured the cell lines by using DMEM with high glucose (4.5 g/L) (44). To support the energy requirement of cancer cells to proliferate, the culture media is supplied with hormones, nutrients and growth factors (45), along with 10% heat inactivated FBS. To prevent contamination in cell culture medium, we utilized 1% pencillin (100U/mL) and streptomycin solution (100 μg/mL). To maintain the physiological pH of 37°C, cell lines were maintained in an incubator with 5% CO_2_ supply (46). Once the confluency of 80 to 90% reached, subculturing was carried out using 0.25% trypsin- EDTA (GIBCO).

#### 2.5.2 Cytotoxicity evaluation using MTT assay

In order to understand the expression pattern of the identified gene from the *in silico* predictions under the influence of Embelin, we employed the MTT assay to understand the cytotoxic effects on the cell lines. After seeding cells (1 × 10^4^) in a 96-well plate and incubating for 24–48 hours, cells were administered with the various concentration ranges of embelin and further incubated for 24 hours. Later on, MTT solution (0.5 mg/mL) was introduced to the cell culture medium and allowed it for formazan crystal formations for four hours and crystals were dissolved in DMSO, followed by measuring absorbance at 570 nm. The percentage of cell viability and the IC_50_ value were calculated using the following formula:


$$Cell\;Viability\;\left(\%\right)=\left(\frac{Absorbance\;of\;Treated\;Sample}{Absorbance\;of\;Control}\right)\;\times \;100\;$$

#### 2.5.3 Colony formation assay

To understand the influence of embelin on cell lines to form one colony from each cell, we employed a colony formation assay by seeding 1 × 10^4^ HCT116 cells (45) into 6 -well plates. After the formation of cell morphology, cells were treated with a range of embelin (10µM to 60µM) for 72 hours (47). Later on, the cell lines were stained using 500 μL of Coomassie Brilliant Blue for 10 minutes at room temperature followed by PBS (phosphate-buffered saline) wash. The stained colonies were observed under white light and photographed for ImageJ software evaluation (48). The following formula was used to determine the percentage survival rate:


$$Survival\;Rate\;\left(\%\right)=\left(\frac{Number\;of\;Colonies\;in\;Treated\;Group\;}{Number\;of\;Colonies\;in\;Control\;Group}\right)\times \;100$$

#### 2.5.4 Cell treatment protocol

The IC_50_ values obtained from the MTT assay were utilized for further analysis. A total of 1 × 10^4^ HCT116 cells were plated in 35mm culture dishes and left to adhere overnight. Once the cells reached the 60% -70% confluency, cells were treated with embelin for 24 h under standard culture conditions (37°C, 5% CO_2_, humidified atmosphere). Simultaneously, another group of cells was treated with 10 μM Oxaliplatin, serving as a positive control due to its known effectiveness as a chemotherapeutic agent in colorectal cancer. An untreated group, which did not receive any drug treatment, was included as the control group for comparative analysis.

#### 2.5.5 Gene expression analysis by quantitative real-time PCR

The total RNA was isolated from both treated and untreated HCT116 cells using the TRIzol-chloroform technique (49). The procedure involved lysing the cells in 1 mL of TRIzol reagent, followed by the addition of 200 μL of chloroform for every 1 mL of TRIzol, and then centrifuging at 12,000 rpm for 15 minutes at 4°C. The RNA-containing aqueous phase was carefully transferred to a fresh tube and precipitated with an equal volume of isopropanol. The resulting RNA pellet was washed with 75% ethanol, allowed to dry, and then dissolved in nuclease-free water. The concentration and purity of the RNA were assessed spectrophotometrically using a Nanodrop at a wavelength of 260/280 nm. cDNA was synthesized using a cDNA conversion kit (TAKARA) and followed by Quantitative PCR analysis employing SYBR Green PCR Master Mix within a real-time PCR system. Primers specific to each gene were designed, and the sequences of the primers are listed in **Table 1**. The PCR cycling conditions involved an initial denaturation step at 95°C for 10 minutes, which was then followed by 40 cycles of a two-temperature regimen: 15 seconds at 95°C, and 1 minute at 60°C. Each experiment was performed in triplicate, and GAPDH was used as the internal control to normalize gene expression. Relative expression levels between control and treated groups were quantified using the ΔΔCt method. Statistical analyses and comparisons among treatment groups were evaluated using a one-way ANOVA to assess overall differences in expression. Data are reported as mean ± standard error of the mean (SEM), and the error bars shown in the graphs represent the SEM calculated from the three biological replicates. Statistical significance was defined as p< 0.05.

**Table 1:** Primers used for gene expression analysis.

Genes	Forward (5’–3’)	Reverse Primer (5’–3’)
BCL2	CTTTTGCTGTGGGGTTTTGT	GTCATTCTGGCCTCTCTTGC
BAX	GGAGCTGCAGAGGATGATTG	CCTCCCAGAAAAATGCCATA
Caspase 3	GGTGCTATTGTGAGGCGGTT	GAGAATGGGGGAAGAGGCAG
COL6A3	GAGACGCAGTGAGTGGGAAA	CAGCCGCACCATTTTTGACA
GAPDH	GACAGTCAGCCGCATCTTCT	GCGCCCAATACGACCAAATC
PI3KCA	GGACCCGATGCGGTTAGAG	ATCAAGTGGATGCCCCACAG

## 3 Results

### 3.1 Co-expression network construction and identification of key modules associated with colon cancer

**Figure 2:**
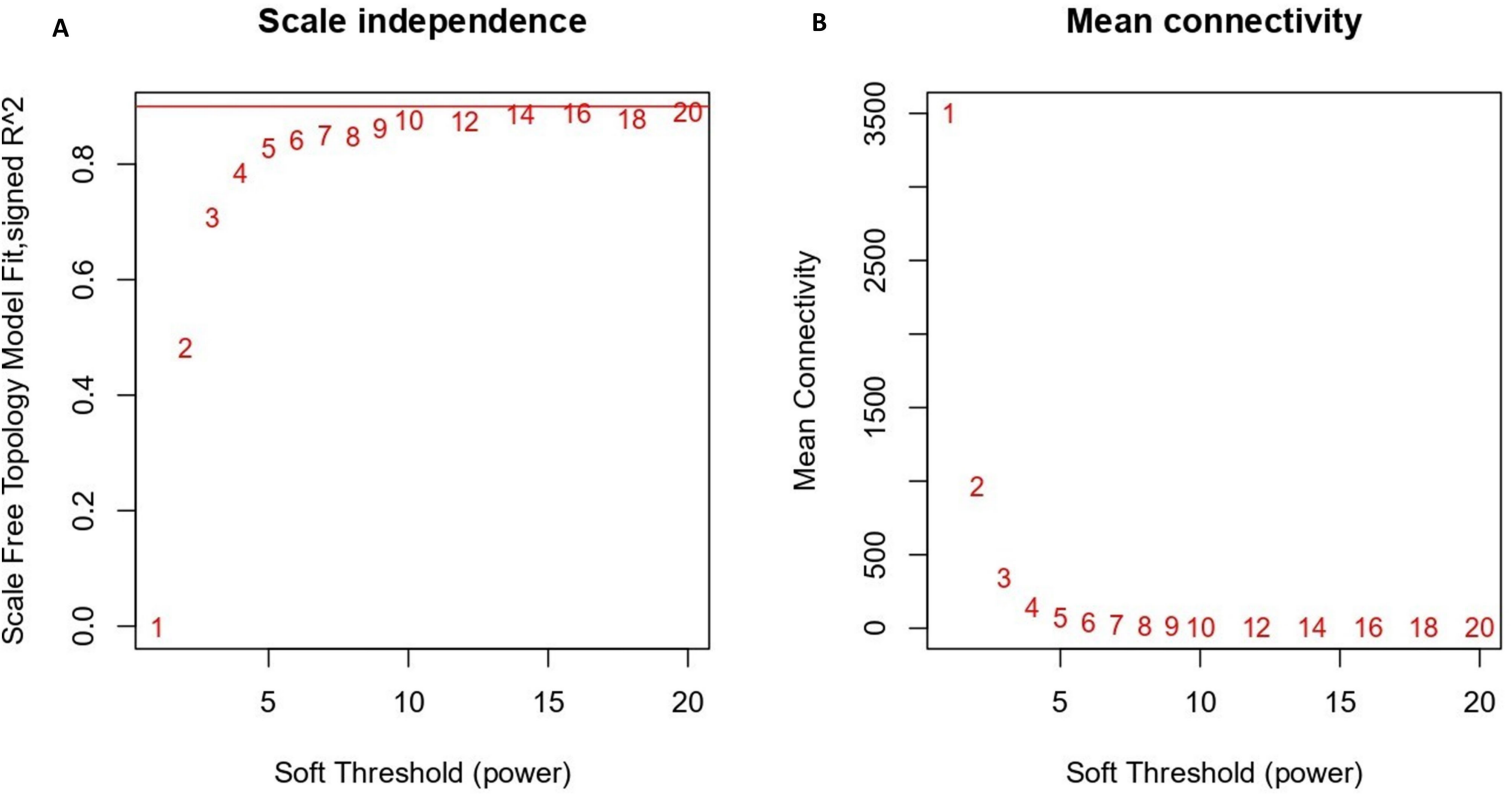
Determination of the optimal soft-thresholding power for WGCNA. **(A)** Scale-free topology fit index (R²) plotted against soft-threshold powers **(B)**. Mean connectivity decreases with increasing power, reflecting stronger gene-gene relationship prioritization.

WGCNA was performed to find gene modules linked to colon cancer using the expression matrix from the GEO dataset (GSE44076), which comprises 246 samples (both healthy and tumor samples) and metadata like gender, age, and sample type. The data were subjected to log2 transformation and RMA normalization before the WGCNA analysis. The primary aim of this study was to identify the prognostic biomarker and target modulated by embelin to treat colon cancer. The samples were divided into two groups (healthy and tumor), and the dataset was screened for outliers and found that there were no significant outliers identified; thus, all samples were retained for subsequent network construction. To ensure that the co-expression network maintained a scale-free topology, a range of soft threshold powers from 1 to 20 was assessed. **Figure 2A** shows the connection between soft-thresholding power and scale-free topology fit. The fit index increases with the rise in power, stabilizing at approximately power 6 when R2 exceeds 0.9, thereby indicating a robust scale-free topology. As power increases, mean connectivity decreases, indicating that stronger gene-gene relationships were prioritized (**Figure 2B**). Power 6 was found to be the ideal soft-thresholding value for building the adjacency matrix and locating co-expression modules in the gene expression data. This value allowed for scale-free topology (R^2^ > 0.85) by preserving sufficient mean connectivity.

**Figure 3:**
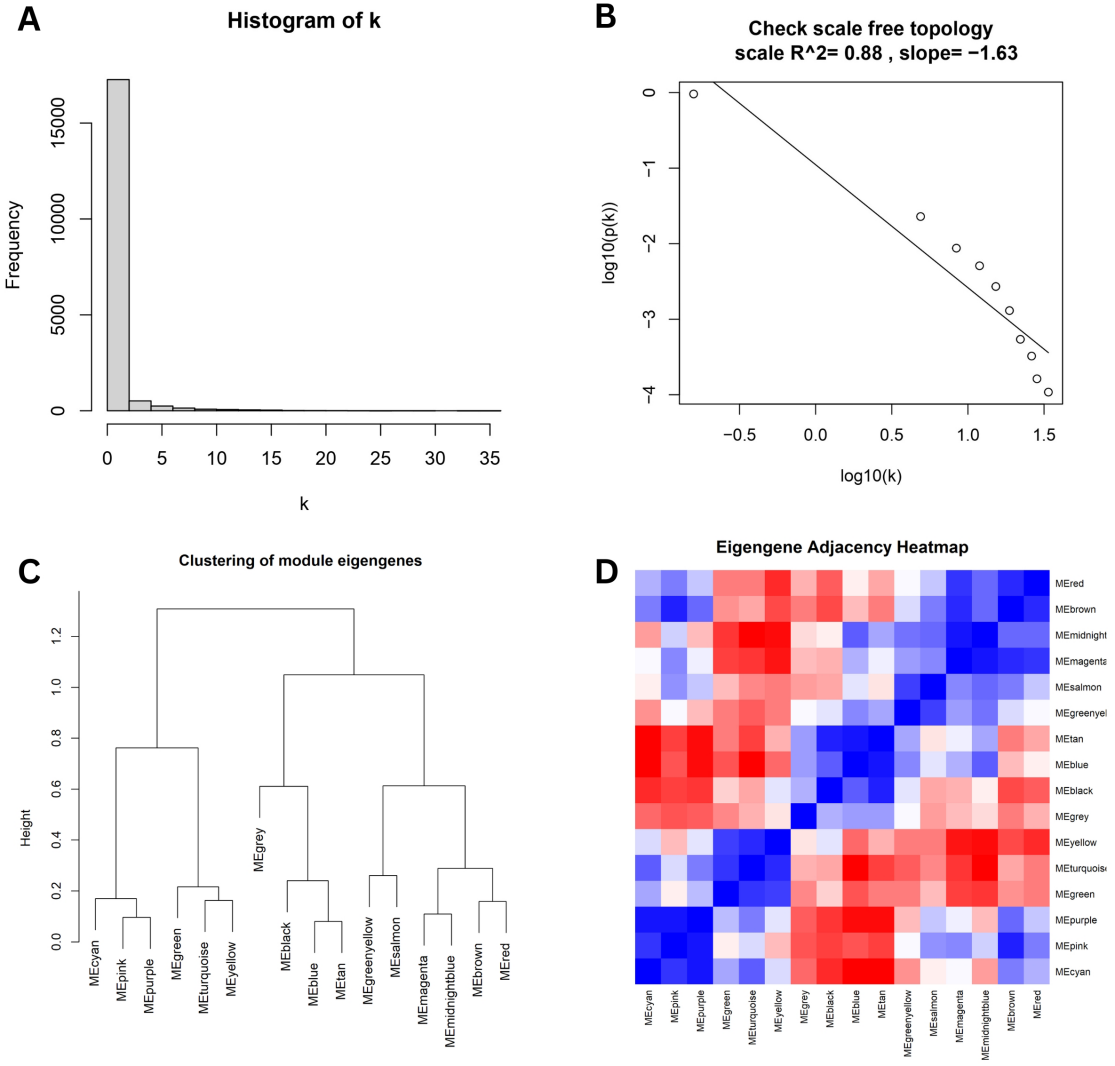
Evaluation of network properties and module relationships in WGCNA: **(A)** Histogram of node connectivity (k) illustrating a right-skewed distribution, **(B)** Log–log plot confirming scale-free topology, **(C)** Dendrogram depicting hierarchical clustering of module eigengenes, **(D)** Heatmap of eigengene adjacency.

**Figure 4:**
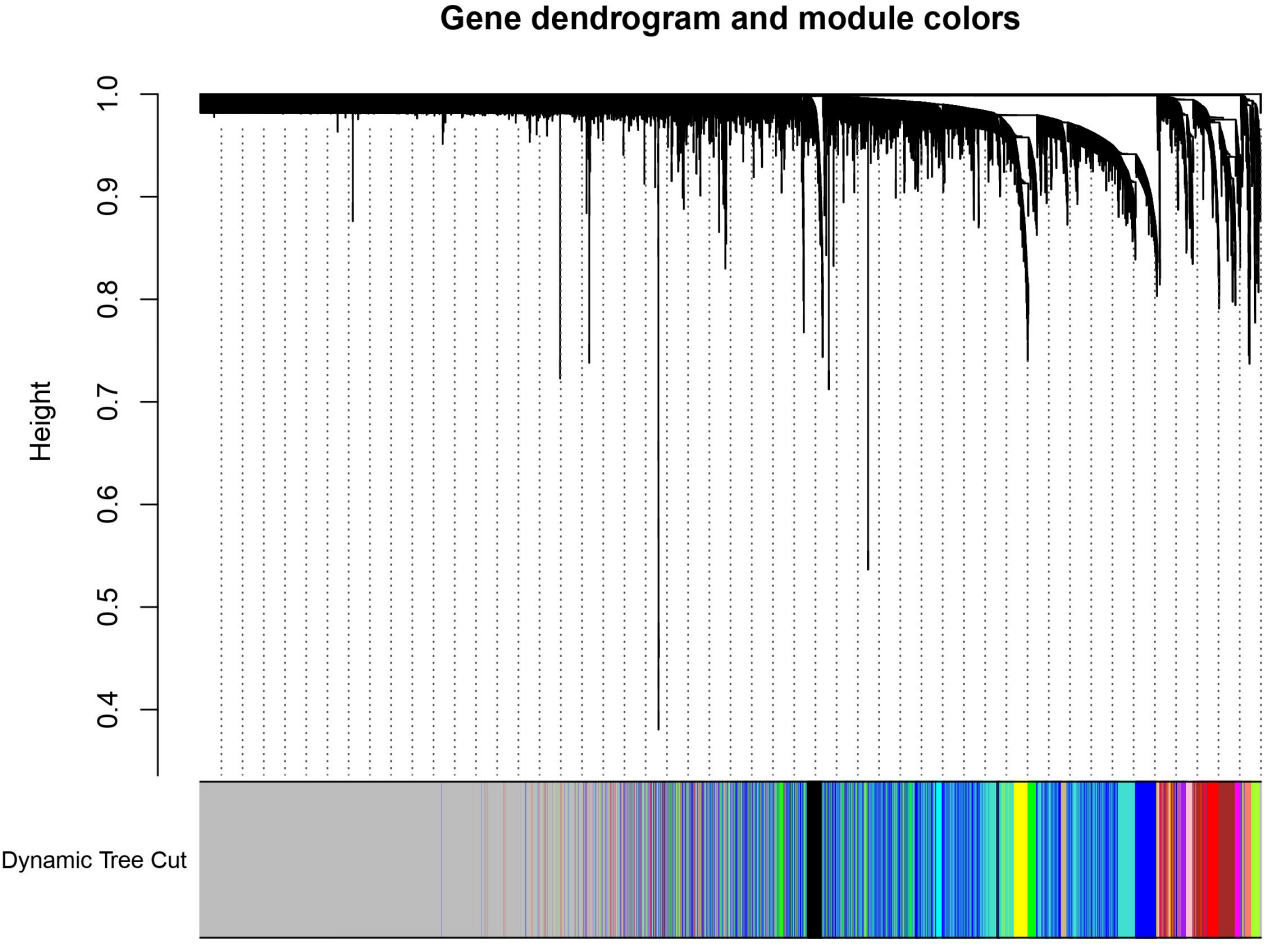
Dendrogram of Hierarchical clustering detected by dynamic tree cut function of WGCNA.

In order to ensure the scale-free topology of the gene co-expression network, we examined diagnostic metrics from WGCNA **Figure 3**. This figure shows a long-tailed pattern in the node connectivity distribution, with most genes showing low connectedness and a small number acting as hub genes, typical of scale-free networks. The log-log plot in **Figure 3B** shows a linear correlation (R^2^ = 0.88) with a negative slope of –1.63, supporting this topology and validating the scale-free structure. We looked at the module eigengenes after identifying the modules using dynamic tree cutting. Modules in **Figure 3C** were arranged based on similar hierarchical clustering dendrogram expression patterns. The correlations between the module eigengenes are depicted in the eigengene adjacency heatmap (**Figure 3D**), where blue denotes negative correlations and red denotes positive correlations. These results confirm the stability of the network and the biological significance of the modules. To illustrate the grouping of genes with similar expression profiles, a hierarchical clustering dendrogram was constructed utilizing the dissimilarity of the topological overlap matrix (TOM) (**Figure 4**). Genes were organized into distinct modules through dynamic tree cutting, each represented by a unique color.

**Figure 5:**
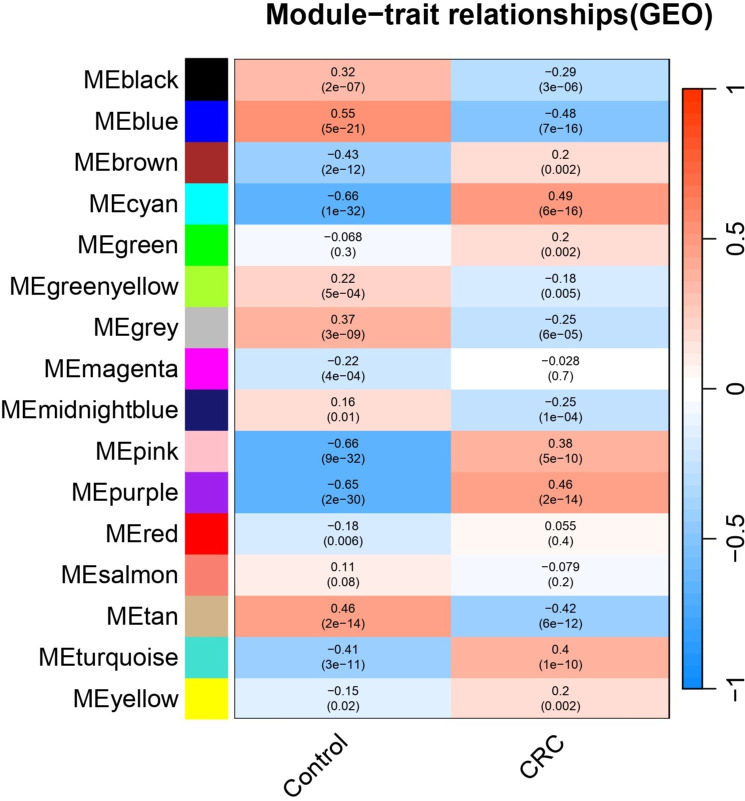
Module–trait relationship of eigengenes with colon cancer.

The application of the dynamic tree cutting method identified multiple co-expression modules, and the dendrogram exhibited a well-organized branching pattern, indicating the presence of closely co-regulated gene groups within the dataset. The heatmap depicting module-trait relationship provides valuable insights into the correlations between gene co-expression modules and phenotypic traits, such as control and colorectal cancer (CRC) conditions (**Figure 5**).

The prominent modules were selected based on the p >0.5 cutoff criteria. Based on the correlation analysis between the module eigengenes under this cutoff, cyan and purple modules were selected. The traits show cyan and purple modules are correlated with the biological relevance in CRC. The positive correlation of cyan (r = 0.49, p = 6^e–16^) and purple (r = 0.46, p = 2^e–14^) modules displays that the genes within these modules have roles in colon cancer progression.

### 3.2 Integration of machine learning and WGCNA results

In this *in silico* study, we combined the Machine learning and WGCNA approaches to obtain a reliable biomarker for colorectal cancer. After training the predictive AI model with training datasets by using three algorithms (LASSO, SVM-RFE and RF), the trained models were used to predict the prominent gene from the expression matrix of the selected modules from WGCNA as input data for the machine learning predictions. To ensure the model accuracy, the training dataset was split into a 70:30 ratio, and the cross-validation strategy was applied to assess the model performance. The results showed higher predictive accuracy with AUC values > ~0.90 (LASSO: 0.87, SVM-RFE: 0.90, RF:0.93), suggesting the capability of the trained model in differentiating the normal and tumour cells.

**Figure 6:**
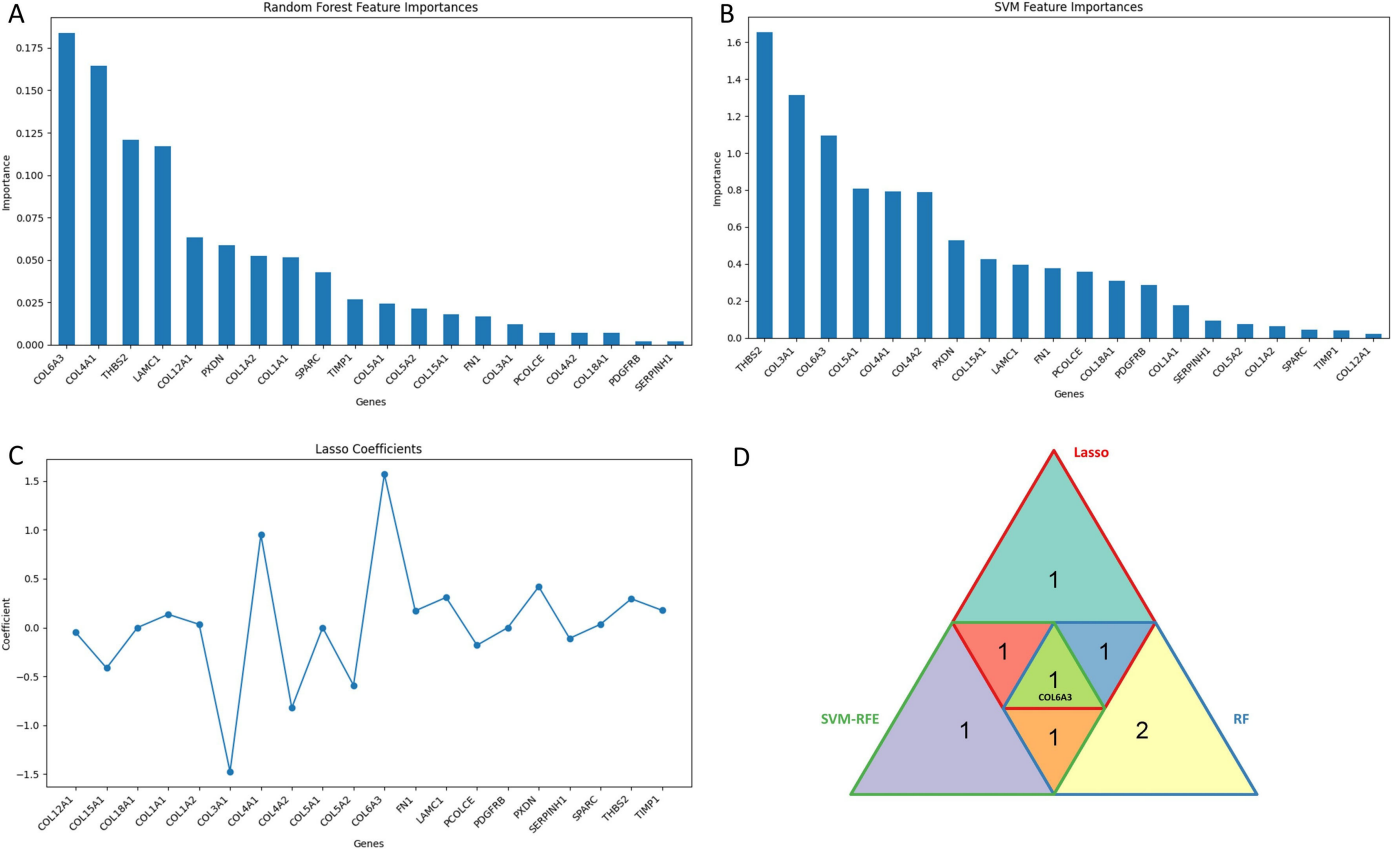
Machine learning-based identification of key genes associated with colon cancer pathogenesis: **(A)** Bar plot of top-ranked genes by feature importance scores from the Random Forest model. **(B)** Feature importance scores from SVM-RFE model **(C)**, Gene coefficients from LASSO regression **(D)** Triangular diagram depicting the overlap among the top five genes identified by each algorithm, highlighting COL6A3 as a common gene in all three algorithms.

The trained models were utilized on a secondary test, WGCNA-derived prediction dataset. Module–trait correlation analysis revealed that the cyan and purple modules exhibited the strongest correlation with colon cancer status. To evaluate predictive relevance, the pre-trained models were utilized on gene expression data from these modules. Genes were ranked based on feature importance scores, and gene expression data were predicted by the trained models. Utilising Gini-based importance measures, the Random Forest (RF) model (**Figure 6A**) identified COL6A3, COL1A1, THBS2, LAMC1, and COL12A1 as the most important genes. These genes are implicated in the tumor microenvironment and metastasis due to their association with the organization and remodeling of the extracellular matrix (ECM). Whereas, the SVM-RFE model also picked ECM-related genes such as THBS2, COL3A1, COL6A3, COL5A1, and COL1A1(**Figure 6B**). LASSO regression, which penalizes redundant predictors and emphasizes sparsity, identified COL6A3, COL5A1, FN1, LAMC1, and COL1A1 as the most significant based on non-zero coefficient values (**Figure 6C**). The top five genes (**Table 2**) from each algorithm were utilized to construct a triangular overlap diagram (**Figure 6D**). COL6A3 was selected by all three models, underscoring its reliability as a potential biomarker and therapeutic target. Genes such as THBS2, COL1A1, COL5A1, and LAMC1 were shared by two algorithms, indicating their potential involvement. Integrating these machine learning methods and the consistent identification of COL6A3 across models highlights its role in matrix dynamics and necessitates further experimental validation.

**Table 2:** Top 5 ranked genes identified by LASSO, SVM-RFE, and random forest algorithms.

Rank	Random forest	SVM-RFE	LASSO
1	**COL6A3**	THBS2	**COL6A3**
2	COL1A1	COL3A1	COL15A1
3	THBS2	**COL6A3**	COL18A1
4	LAMC1	COL5A1	COL1A1
5	COL12A1	COL4A1	COL11A2

COL6A3 a common gene ranked by all three algorithms.

### 3.3 Cross-validation of gene targets identified by machine learning algorithms

**Figure 7:**
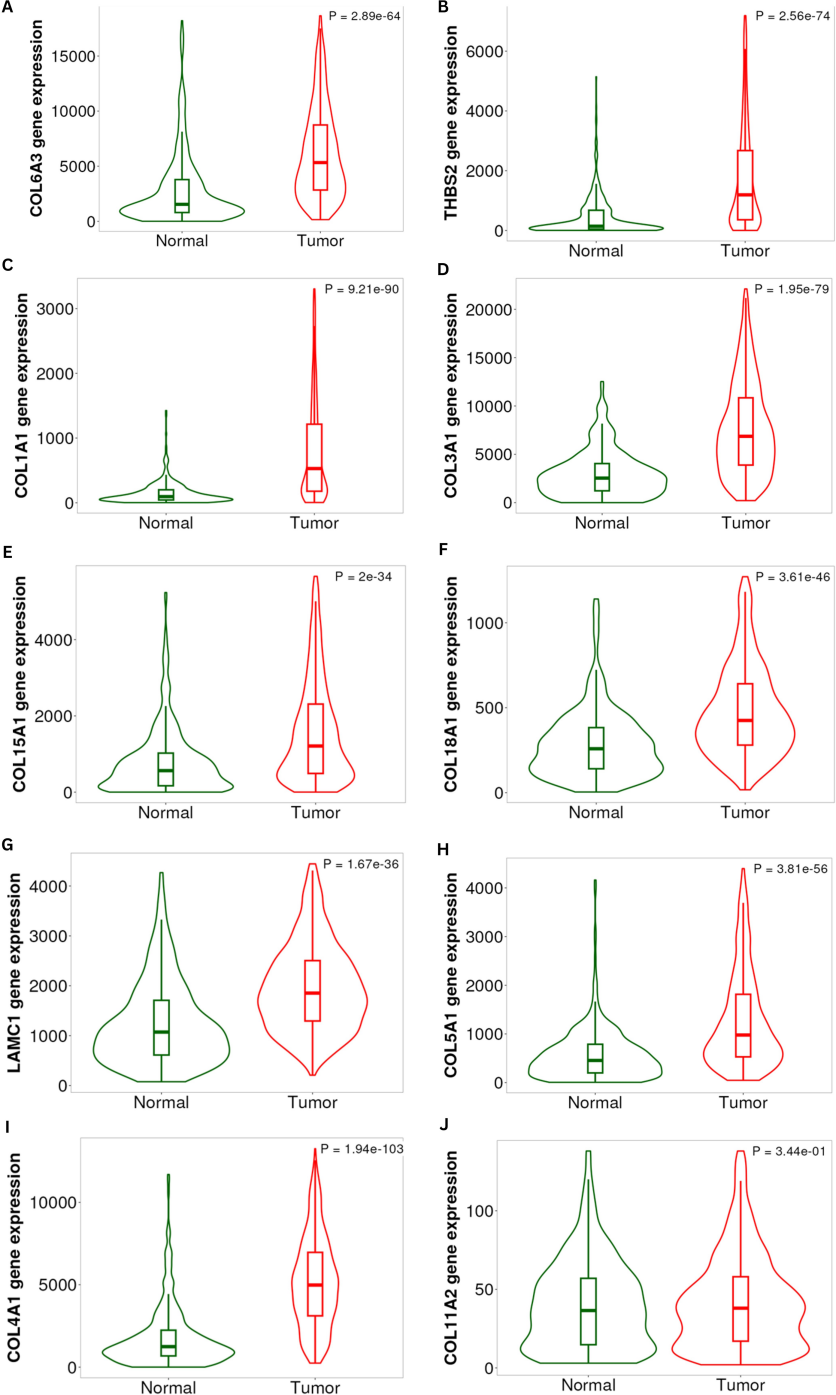
Differential expression of top 5 ranked genes from all three algorithms between normal (green) and tumor (red) samples: **(A)** COL6A3, **(B)** COL1A2, **(C)** COL1A1, **(D)** COL3A1, **(E)** COL5A1, **(F)** COL4A1, **(G)** LAMC1, **(H)** COL8A1, **(I)** COL4A3, and **(J)** COL11A1. Boxplots embedded within violins indicate median and interquartile ranges. Statistical significance between normal and tumor groups is shown as P values in each panel.

**Figure 8:**
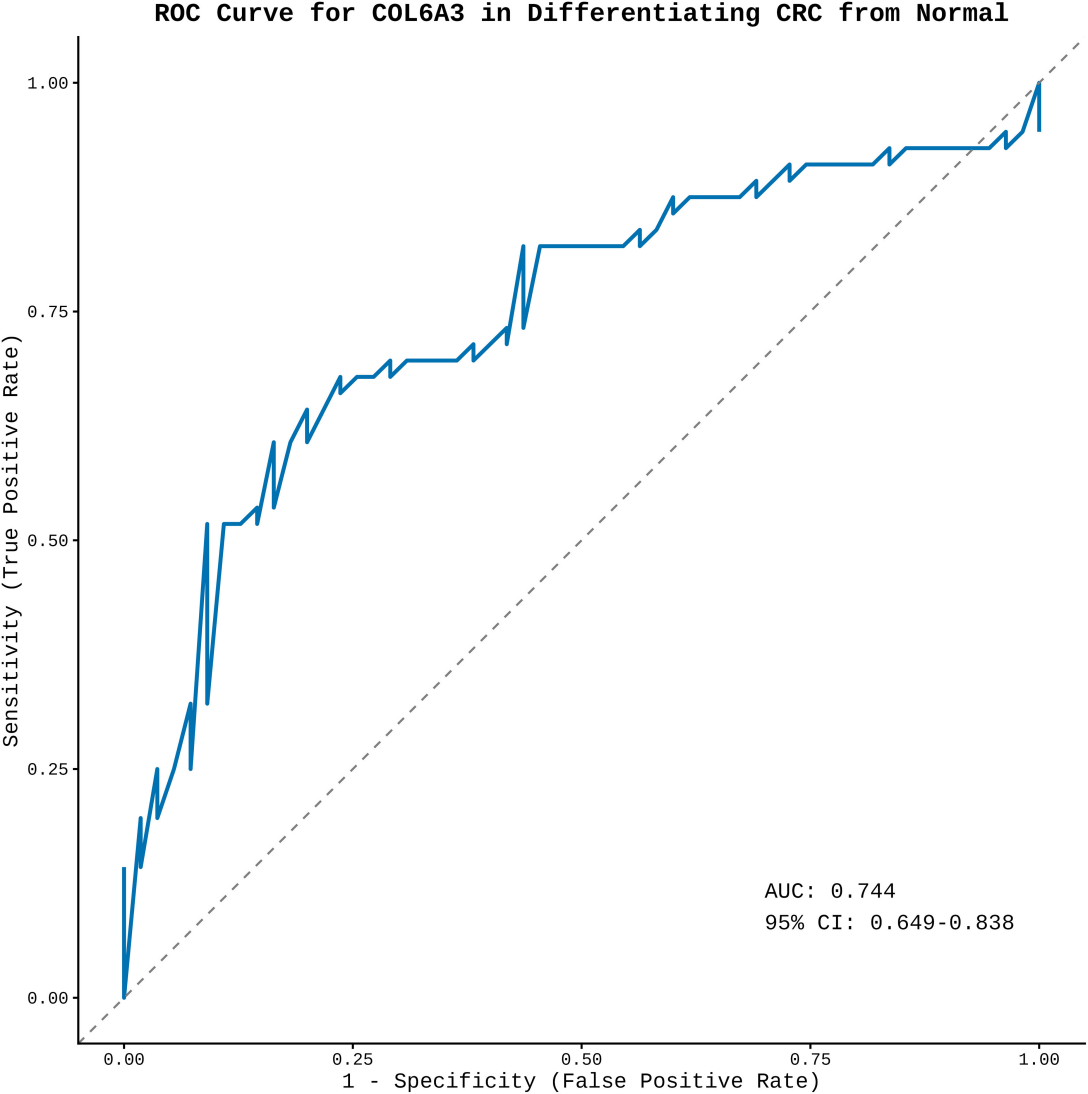
Receiver Operating Characteristic (ROC) Curve showing capability of COL6A3 in differentiating healthy groups from tumour groups.

To enhance the biological validity and reliability of candidate gene targets predicted and ranked by machine learning methods such as LASSO, Random Forest, and SVM-RFE, we performed cross-validation using the external transcriptomic dataset (GSE44861) as a validation dataset, which comprises gene expression profiles from both normal (n = 55) and tumour colon tissues (n =56), making it suitable for validating predictions from trained models. We pre-processed the data through background correction, quantile normalization, and log_2_ transformation to ensure data consistency and reduce noise. The expression patterns identified by the model, highlighting genes in both the validation dataset and between colon tumour and normal tissues, were illustrated in **Figure 7**. To further confirm expression patterns and considering the sample size of the validation dataset, we utilized the UALCAN portal, an online resource that leverages expression data from the TCGA repository. The expression patterns, analyzed based on gene symbols and the colon cancer dataset, were presented in **Supplementary Figure 1**. In colon cancer tissues, cross-validation consistently revealed high expression levels of several extracellular matrix (ECM)-associated genes. COL6A3, COL1A1, COL3A1, THBS2, COL5A1, and LAMC1 all exhibited significant upregulation with highly significant p-values (e.g., COL6A3, p = 2.89^e-64^; COL1A1, p = 9.21^e-90^) (**Figure 7A**). To understand the distinction between healthy and tumour cells of COL6A3 which was commonly predicted by all three algorithms, we employed ROC and AUC analysis using expression profile of COL6A3 in validation dataset. The AUC curve (0.744) showed a value greater than 0.5, and a higher confidence interval (CI) (0.649-0.838), indicative of the distinguishing capacity of COL6A3 and the diagnostic accuracy of this gene in colorectal cancer (**Figure 8**).

COL6A3 was consistently ranked by all three algorithms as a significant component of the extracellular matrix (ECM) and pro-oncogenic signalling pathways. Its overexpression was correlated with poor prognosis and contributes to cell survival, proliferation, and resistance to apoptosis. Additionally, COL1A1 and COL3A1, which encode other fibrillar collagens, play a role in ECM remodelling and facilitate tumour invasiveness. These genes play a major role in oncogenic signalling, such as FAK/Src, TGF-β, and PI3K/AKT. The overexpression of all these genes promotes the tumour stemness, epithelial-mesenchymal transition (EMT), along with chemotherapy resistance. Their consistent overexpression in tumors and identification as ML-prioritised hub genes underscores their promise as diagnostic and prognostic biomarkers in colon cancer. Other than the collagen genes, ML algorithms also ranked the MMPs-associated genes, such as THBS2, the thrombospondin gene significantly upregulated in tumour tissue (p = 2.56^e-74^). The overexpression of THBS2 promotes tumor progression via VEGF and Notch oncogenic pathways. Other than these genes, ML also ranked LAMC1, COL5A1, and COL15A1 genes, which also contribute to ECM-receptor interactions and oncogenic pathways by enhancing the cell adhesion, migration, and invasiveness of the tumor cells. COL11A2, on the other hand, did not exhibit a statistically significant difference in expression between normal and tumour tissues (p = 3.44^e-01^). This suggests that the gene was either less important in the progression of colon cancer or that its dysregulation was specific to a particular context or subtype. The specificity of other ECM-related genes as reliable, repeatable markers across datasets was supported by this contrast. The validity of ECM-related genes as molecular markers of colon cancer was supported by their consistent overexpression across separate TCGA datasets. These genes are promising biomarkers for diagnosis, prognosis, and therapeutic stratification in addition to being downstream effectors of oncogenic signalling pathways like integrin-mediated FAK-Src signalling, TGF-β, MAPK, and PI3K/AKT.

COL6A3 has been selected as the primary candidate for *in vitro* validation due to its major role within the ECM-associated gene network, as identified by machine learning, and its consistent and significant upregulation across many independent datasets. Of all candidate genes, COL6A3 had shown the most statistically significant tumour vs normal tissue expression differences. It is a critical node within networks related to the aetiology of colon cancer, the PI3K/AKT pathway. Its involvement in tumour invasion, ECM reorganisation, and resistance to apoptosis underlines its biological relevance and translational potential. Focusing on COL6A3, we intend to validate a target with therapeutic potential as a biomarker.

### 3.4 Cytotoxic effects of the compound assessed by MTT assay

**Figure 9:**
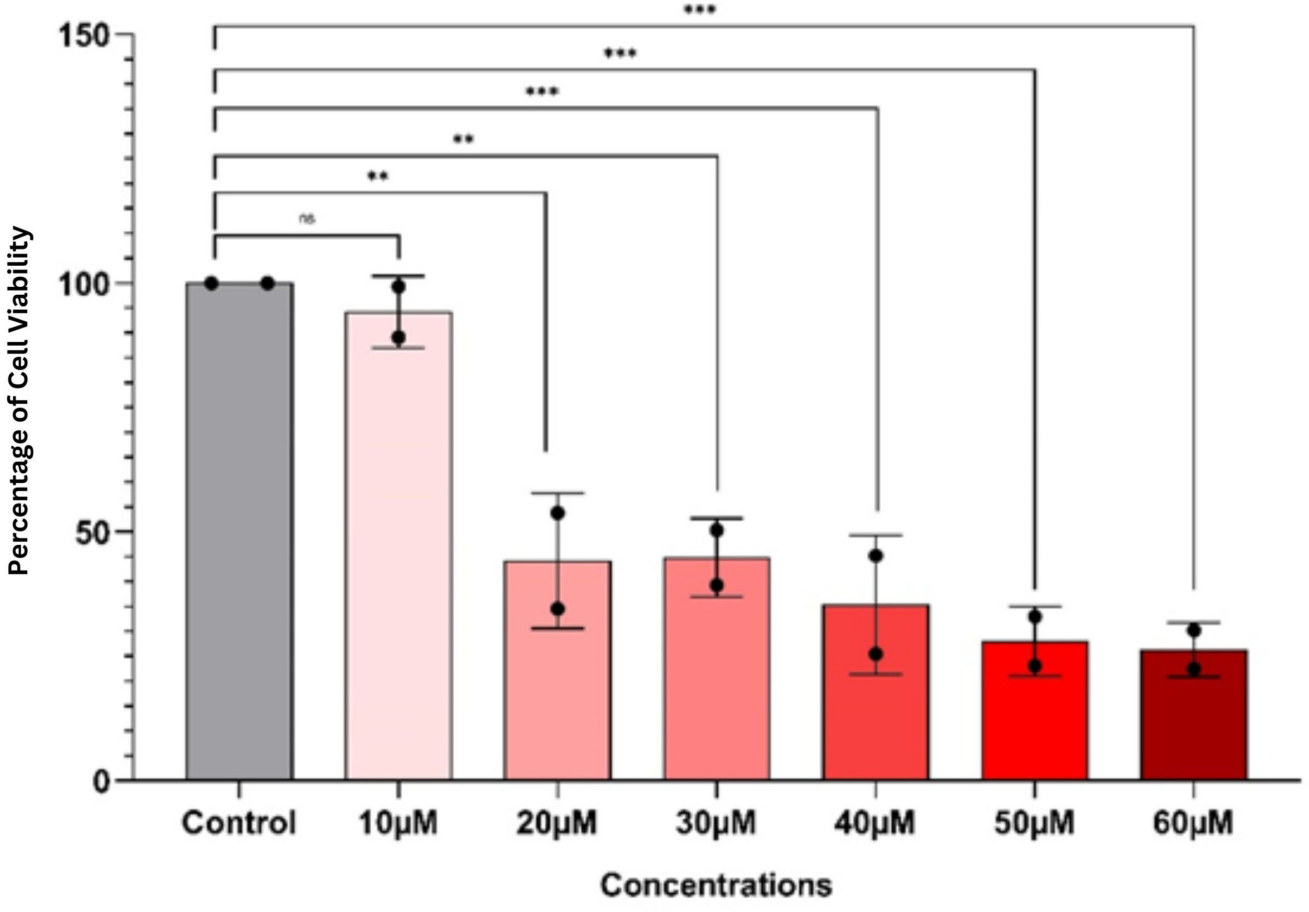
MTT assay showing the dose-dependent effect of the compound on colon cancer cell viability. Bars represent mean ± SD of three biological replicates. Statistical significance compared to the control group is indicated as: ns = not significant; ** = p < 0.01; *** = p < 0.001.

The cytotoxic effects of HCT116 cells were assessed using the MTT assay. Cells were treated with embelin at various concentrations (10–60 μM). The assay results showed that the viability of the cells was lower at 10 μM, with no statistical significance, whereas at 20 μM (p< 0.01), concentration, we found a significant decrease in cellular viability with higher statistical significance, followed by 30 μM (p< 0.001). Notably, we seen the plateau of cytotoxic effect of embelin between 30 μM and 60 μM (**Figure 9**). These findings suggest that the compound exerts a significant cytotoxic effect on colon cancer cells, ranging between 20 μM and 30 μM, with maximum effectiveness achieved around 30 μM, beyond which no substantial further decrease in viability occurs.

### 3.5 Dose-dependent colony inhibition potential of embelin

Colony formation assay was performed to assess the long-term anti-proliferative effect of embelin on HTC-116 colon cancer cells with treatments spread over 10 to 60 μM. This assay serves as a backup to the viability tests described in MTT and tries to confirm if the surviving cells in MTT can continue proliferating indefinitely to form colonies. Colonies in control and DMSO-treated groups were densely packed and were morphologically distinct and well-shaped, suggesting that DMSO did not induce any cytotoxicity (**Supplementary Figure 2**). Counting of the number of colonies, as well as the number of colonies on the plates, showed that the number of colonies decreased in a dose-dependent manner. Colony formation was significantly inhibited at 30 µM and above, and there was a noticeable reduction in colony numbers from 20 µM. Quantitative analysis of colony area and density demonstrated that cell viability decreased to 68.60% at 20 µM and further to 64.90% at 30 µM, compared to the untreated control, indicating a significant early inhibition of colony formation (**Supplementary Table 1**). At concentrations ranging from 40 µM to 60 µM, colony formation was significantly impaired, indicating significant cytostatic effects within this range. Thus, the MTT assay outcomes show that the IC_50_ value of embelin is between 20 µM and 30 µM to prevent colon cancer progression in lower concentrations. These results show the potential of embelin cancer treatments at lower concentrations.

### 3.6 The role of COL6A3 in PI3K/AKT signaling pathway

**Figure 10:**
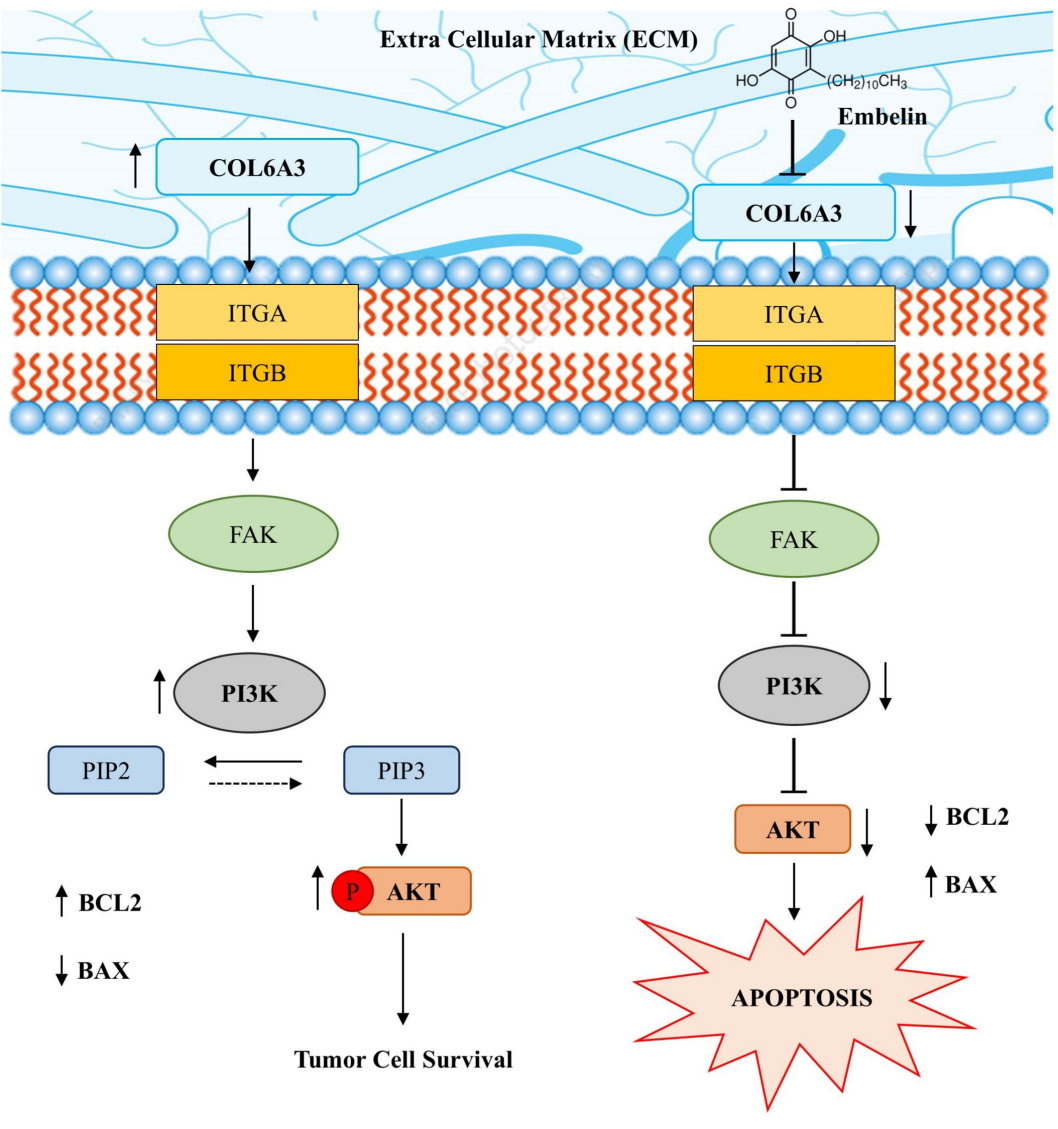
Mechanism of action of Embelin in modulating PI3K/AKT signalling via COL6A3–Integrin axis.

COL6A3 has emerged as a prominent gene from the combination of machine learning and the WGCNA approach and validated through *in vitro* studies as well. COL6A3 stands out as a prominent target for colon cancer treatments because of its association with the PI3K/AKT signalling pathway. The pathway through which COL6A3 evades the colon cancer progression is depicted in **Figure 10**. Many studies shown that the overexpression of COL6A3 in various types of cancer increases the metastasis potential of cancer cells.

The interaction between the COL6A3 and the transmembrane receptors (ITGA/ITGB) triggers the activation of focal adhesion (FAK), thus FAK activates the PI3K, which in turn phosphorylates AKT and activates AKT to convert PIP2 into PIP3. This cascade is very much essential for a tumour cell for the continuous cellular proliferation and survival by inhibiting the apoptosis pathways, which downregulates the pro-apoptotic protein (BAX) by elevating the anti- apoptotic protein (BCL2). Whereas in this study, we have shown that the expression COL6A3 is significantly reduced upon administration of embelin by weakening the integrin-mediated signalling. We propose the mechanism of action of embelin via the putative mediator COL6A3 by modulating Intergin-FAK-PI3K pathway by downregulating the BAX levels and elevating the BCL2 levels in the colorectal cancer cells.

### 3.7 Expression profiling of PI3K/AKT pathway–associated apoptotic genes following embelin treatment

We performed the gene expression analysis to support our hypothesis that the embelin modulates the apoptosis signalling by interfering with integrin mediated PI3K/AKT mediated signalling pathway. Thus, the prominent genes identified were COL6A3, Caspase3, BCL2, BAX, and PI3KCA. These genes were chosen based on our computational analysis, which identified COL6A3 as a critical target involved in tumorigenic signalling through modulation of the PI3K/AKT pathway. Embelin was administered to HCT116 colorectal cancer cells for 24 hours at concentrations of 20 µM and 30 µM, derived from our prior cytotoxicity screening using MTT and colony formation assays, to evaluate downstream effects. Gene expression levels were compared to those of a positive control group (cells treated with 10 µM oxaliplatin) and an untreated control.

**Figure 11:**
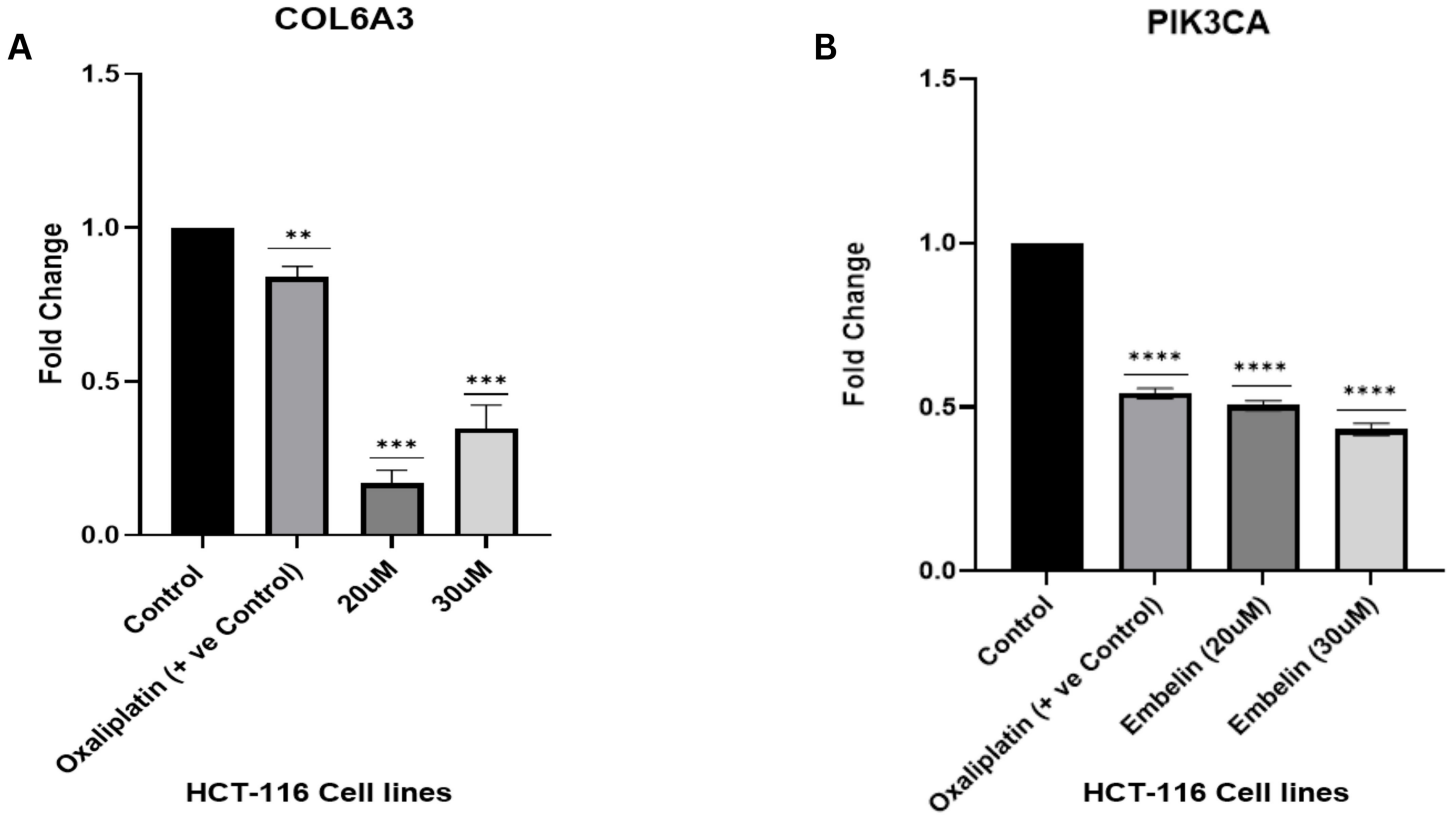
Gene Expression analysis: **(A)** Relative mRNA expression levels of COL6A3 following treatment with Oxaliplatin (positive control) and Embelin (20 µM and 30 µM), **(B)** Relative expression levels of PIK3CA. Asterisks indicate levels of statistical significance compared to a control group. **, p < 0.01; ***, p < 0.001; ****, p < 0.0001. The results marked with more asterisks have a stronger statistical significance.

To validate the repression of target gene expression observed in our functional and in silico analysis, qRT-PCR was used to examine the expression of COL6A3 and PIK3CA in Embelin-treated colon cancer HCT-116 cells at two drug concentrations (20 µM and 30 µM). Oxaliplatin served as a positive control. **Figure 11A** showed that COL6A3 expression was significantly reduced after treatment. Compared to the untreated control (fold change = 1), Oxaliplatin caused a modest but significant decrease (p< 0.01), while embelin produced more potent suppression of gene expression. Specifically, 20 µM embelin lowered COL6A3 expression to below 0.3-fold (p< 0.001), and 30 µM, despite a weaker stimulation than 20 µM, still caused significant suppression (p< 0.001) with a dose-dependent effect. Additionally, embelin significantly reduced PIK3CA expression (**Figure 11B**). The expression level of PIK3CA was significantly reduced across oxaliplatin and embelin treatment groups, whereas downregulation of PIK3CA was significant at 30 µM Embelin. These results showed that the expression level of COL6A3 and PIK3CA gene was significantly modulated by embelin demonstrating its potential to curb the colorectal cancer.

**Figure 12:**
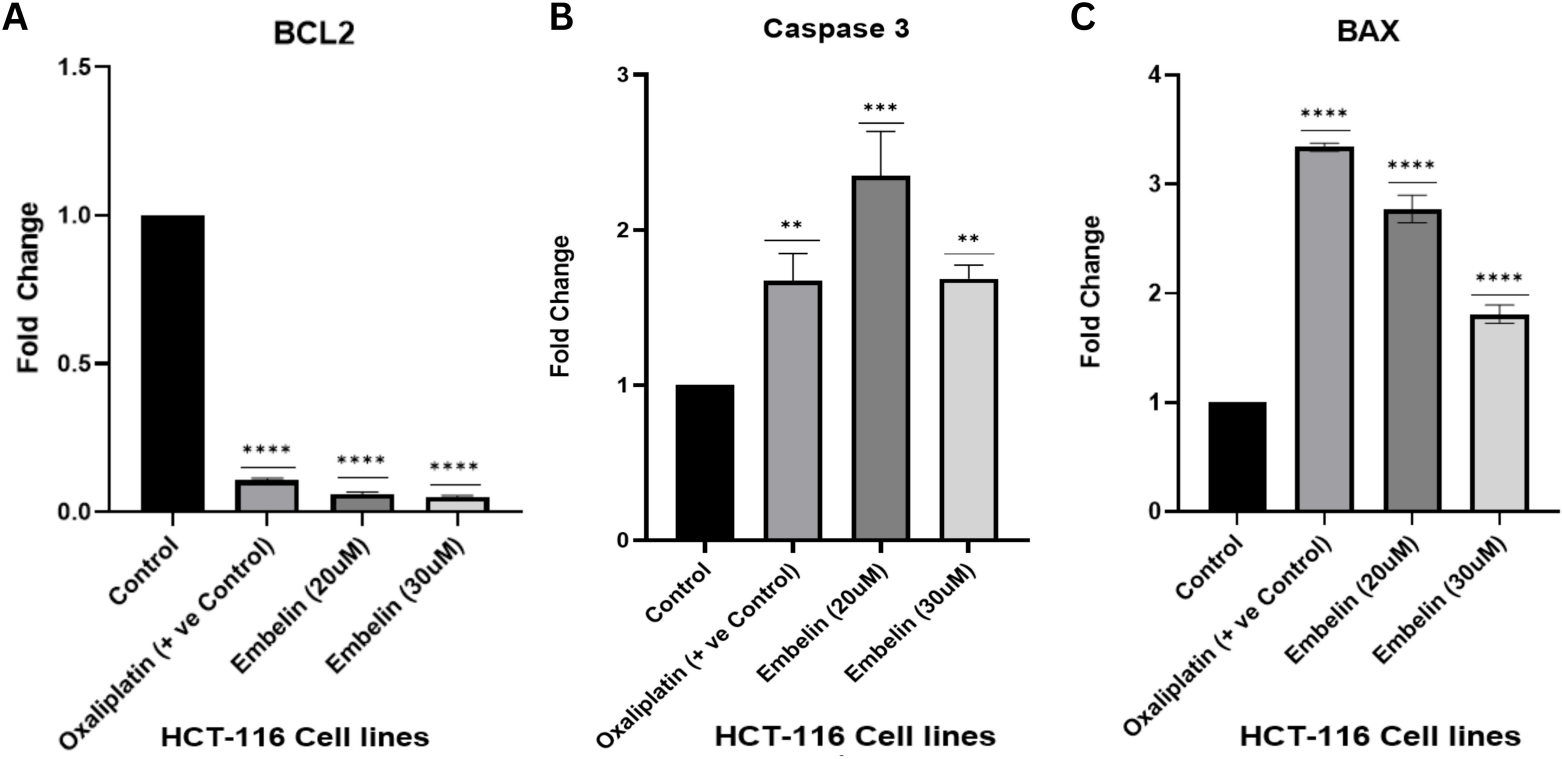
Gene expression analysis of PI3K/AKT pathway–related apoptotic markers: **(A)** BCL2 **(B)** Caspase 3, **(C)** BAX. Asterisks indicate levels of statistical significance compared to a control group. **, p < 0.01; ***, p < 0.001; ****, p < 0.0001. The results marked with more asterisks have a stronger statistical significance.

Post-treatment quantitative expression analysis revealed a significant downregulation of the anti-apoptotic gene BCL2 (**Figure 12A**). Oxaliplatin and embelin (20 µM and 30 µM) substantially suppressed anti-apoptotic signaling, reducing BCL2 expression by approximately 90% (0.1-fold of control). This change was highly statistically significant compared to control (**** p< 0.0001) and indicated a saturation point in BCL2 inhibition. These results further support the hypothesis that embelin effectively inhibits the PI3K/AKT signaling pathway at the transcriptional level. Interestingly, the pro-apoptotic gene Caspase 3 was most induced at 20 µM Embelin treatment, with a fold change of about 2.5, which exceeds the response seen with Oxaliplatin (~1.2-fold). A slight decrease to around 1.8-fold was observed at 30 µM,

potentially indicating feedback inhibition or cytotoxic stress at higher doses (**Figure 12B**). Consistent with studies showing dose-dependent caspase kinetics during apoptosis, this trend suggests that 20 µM embelin may be the optimal concentration for inducing apoptosis via Caspase 3 activation. Finally, across all treatment conditions, there was an increased expression of BAX, a key pro-apoptotic gene antagonistic to BCL2. Oxaliplatin elicited the strongest response (~3.3-fold), followed by 20 µM Embelin (~3.0-fold), and 30 µM Embelin (~2.0-fold) (**Figure 12C**). Collectively, these observations support that embelin effectively suppresses the PI3K/AKT pathway by increasing pro-apoptotic gene expression, such as Caspase 3 and BAX, while simultaneously downregulating pro-survival markers like BCL2 and PIK3CA. This expression pattern reinforces the modulatory potential of embelin on key apoptosis regulators in cancer signaling pathways, thus underpinning the mechanistic hypothesis proposed in **Figure 10**.

## 4 Discussion

Colorectal cancer (CRC) is one of the dreadful diseases causing death worldwide. CRC originates from the epithelial lining of the colon to the rectum. The pathogenesis of colon cancer is influenced by a multitude of factors, including physiological and epigenetic elements. Despite advancements in therapeutic strategies, a significant number of patients are diagnosed only at advanced stages, where the prognosis remains poor due to metastasis and resistance to therapy, and managing colorectal cancer remains challenging. A significant hurdle is the lack of reliable and specific biomarkers for early detection, along with the need for less toxic and dependable small molecules to prevent drug resistance and minimize chemotherapeutic side effects. The recent advancements in systems biology and computational techniques, along with the use of multi-omics high-throughput omics data, provide a robust approach to discover critical genes in the pathogenesis of colon cancer. The combination of a multi-omics approach with machine learning algorithms enhances reliable biomarker discovery, which could facilitate treatment options that improve patient survival and quality of life. Natural compounds are increasingly gaining attention in cancer research due to their potential influence on cancer pathways, with safer options and reduced toxicity compared to chemotherapeutic drugs. One such compound from *Embelia ribes* is Embelin, a benzoquinone compound that exhibits anti-inflammatory, antioxidant, and anti-cancer properties. Studies have shown that embelin can also interfere with cancer-promoting pathways such as NF-kB, STAT3, and PI3K/AKT. The ability of embelin to selectively induce apoptosis in cancer cells without damaging normal cells highlights its promise as a candidate for cancer treatment.

The primary objective of this study is to utilize a combination of machine learning and a multi-omics approach to identify a reliable biomarker and target that can serve as a potential mediator of embelin activity, thereby preventing the progression of colorectal cancer. To understand the pathogenesis of colon cancer, we utilised the system biology approach by integrating the WGCNA on the GEO dataset (GSE44076). Cyan and purple modules from the network are significantly associated with colon cancer, and the genes from these two modules are subjected to gene ontology studies to confirm their role in cancer pathogenesis. The gene expression matrix of these two modules was considered as test data for further predictions from machine learning models. In this study, we employed three major machine learning algorithms: SVM-RFE, LASSO, and Random Forest, due to their potential to distinguish between the healthy and tumour classes. Initially, the models were trained by combining the two different datasets obtained from the GEO database (GSE41258, GSE39582) by extracting gene expression profiles. The criteria for feature selection are healthy and CRC tumour data. The accuracy and cross validation of the trained models suggested that the trained modules were good enough for the predictions. We used the test data (gene expression matrix of cyan and purple modules from the WGCNA) and the trained models predicted a few genes as the prominent genes. After integrating all three algorithms, we identified COL6A3 as the prominent gene across all the algorithms. The cross-validation with an external transcriptomic dataset (GSE 44861) as well as TCGA datasets shows that the upregulation of COL6A3 occurs in tumor cells compared to healthy colon cells. The ROC and AUC analysis suggested that the strong distinguishing ability of normal to tumour colon cells with the expression levels of COL6A3 provides strong support that COL6A3 can be a potential diagnostic biomarker.

Basically, the COL6A3 gene encodes the alpha-3 chain of type VI collagen, a flexible extracellular matrix protein that forms microfibrils and organizes the matrix around cells. COL6A3 contributes to connective tissue structure by combining with other collagen VI chains to form mature collagen molecules. It is expressed in cancer-associated fibroblasts within the tumor microenvironment and influences tumor growth, invasion, and metastasis. COL6A3 has been implicated in osteosarcoma and gastric cancer progression, modulating key signaling pathways such as PI3K/AKT, which are critical for cancer cell survival and motility. The subsequent cleavage of COL6A3 releases the potent signaling peptide Endotrophin (ETP), which can induce Epithelial-Mesenchymal Transition (EMT), enhance metastasis, and promote chemoresistance. This suggests COL6A3 contributes to a tumor-supportive matrix and may serve as a potential therapeutic target. Although COL6A3 is a significant target for preventing cancer progression, there are no approved drugs that can directly inhibit it. Most therapeutic strategies aim to disrupt the extracellular matrix (ECM)-driven signaling pathways. COL6A3 interacts with key downstream components such as α2β1 and α1β1, which initiate the pro-invasive signaling cascade. Efforts have concentrated on inhibiting these pathway components, particularly Focal Adhesion Kinase (FAK), using agents like Defactinib, which functionally suppress ECM-driven signaling. Some strategies have also attempted to halt ECM remodelling by employing matrix metalloproteinase (MMP) inhibitors. However, the clinical success of these agents has been minimal due to their poor efficacy and musculoskeletal toxicities. These challenges in targeting COL6A3-associated pathways underscore the need for more selective intervention strategies.

We evaluated the transcriptional response of key genes aligned with our computational predictions. Our Weighted Gene Co-expression Network Analysis (WGCNA) and machine learning analyses showed that hub gene COL6A3 is associated with genes PIK3CA, BCL2, BAX, and Caspase-3. These genes are critical for the PI3K/AKT pathway and apoptotic signaling. In order to understand the role of phytocompound Embelin on the COL6A3 obtained from *in silico* studies, we leveraged quantitative PCR analysis, which indicated that Embelin inhibited PI3K-mediated survival signaling by downregulating PIK3CA expression in a dose-dependent manner, consistent with the ability of Embelin to inhibit the PI3K/AKT cascade. The expression of BCL2, a critical anti-apoptotic regulator, was significantly reduced (~90%) at both 20 µM and 30 µM concentrations of Embelin, comparable to the suppression observed with the positive control (Oxaliplatin). This suggests a threshold-dependent effect rather than a dose-dependent one, as evidenced by the consistent downregulation of BCL2 at both concentrations. Conversely, following Embelin treatment, the pro-apoptotic markers BAX and Caspase-3 were markedly elevated. Notably, BAX expression peaked at 20 µM, achieving a fold change similar to that of Oxaliplatin, whereas at 30 µM, the increase was somewhat attenuated. Similarly, Caspase-3 expression peaked at 20 µM, with a ~2.5-fold increase, and then slightly decreased at 30 µM, showing a ~1.8-fold decrease. These findings suggest that embelin, at lower concentrations, induces apoptosis, further supporting its role in modulating programmed cell death through the disruption of ECM-integrin-FAK-PI3K/AKT signalling pathways.

## 5 Conclusion

The combination of multi-omics and the machine learning approaches identified COL6A3 as a prominent biomarker and a potential target in colorectal cancer, and a putative mediator of embelin in controlling colorectal cancer progression. The cross-validation studies support the capability of COL6A3 in differentiating the healthy cells from tumour cells. We also proposed the pathway in which COL6A3 has a role with the ECM-intergin-FAK-PI3K/AKT signalling pathway and how downregulation of COL6A3 can induce the apoptosis pathway. Overall, this research underscores the potential of artificial intelligence in facilitating biomarker identification and the development of targeted therapies, thereby paving the way for future translational studies in personalized, ECM-focused cancer treatments.

## 6 Limitations

Despite the promising results from this study, the co-expression network is derived from transcriptomic profiles, necessitating protein-level validation and functional assays, such as knockdown studies of COL6A3, to confirm the mechanistic roles. Furthermore, *in vivo* studies are essential to establish the therapeutic efficacy and specificity of embelin in targeting COL6A3 within tumour microenvironments.

## Data Availability

The datasets utilized in this research were obtained from publicly available databases, including the GEO repository (GSE44076, GSE41258, GSE39582 and GSE44861). Further inquiries can be directed to the corresponding author.
