# Supplementary material for: Artificial intelligence driven multi-omics framework identifies COL6A3 as a diagnostic biomarker and a putative gene target modulated by Embelin in Colorectal cancer
Source: Front Oncol. 2026 Feb 2;16:1711079. doi: 10.3389/fonc.2026.1711079 (PMC12907207; doi:10.3389/fonc.2026.1711079)
Supplement: Supplementary file 1 [file DataSheet1.pdf]

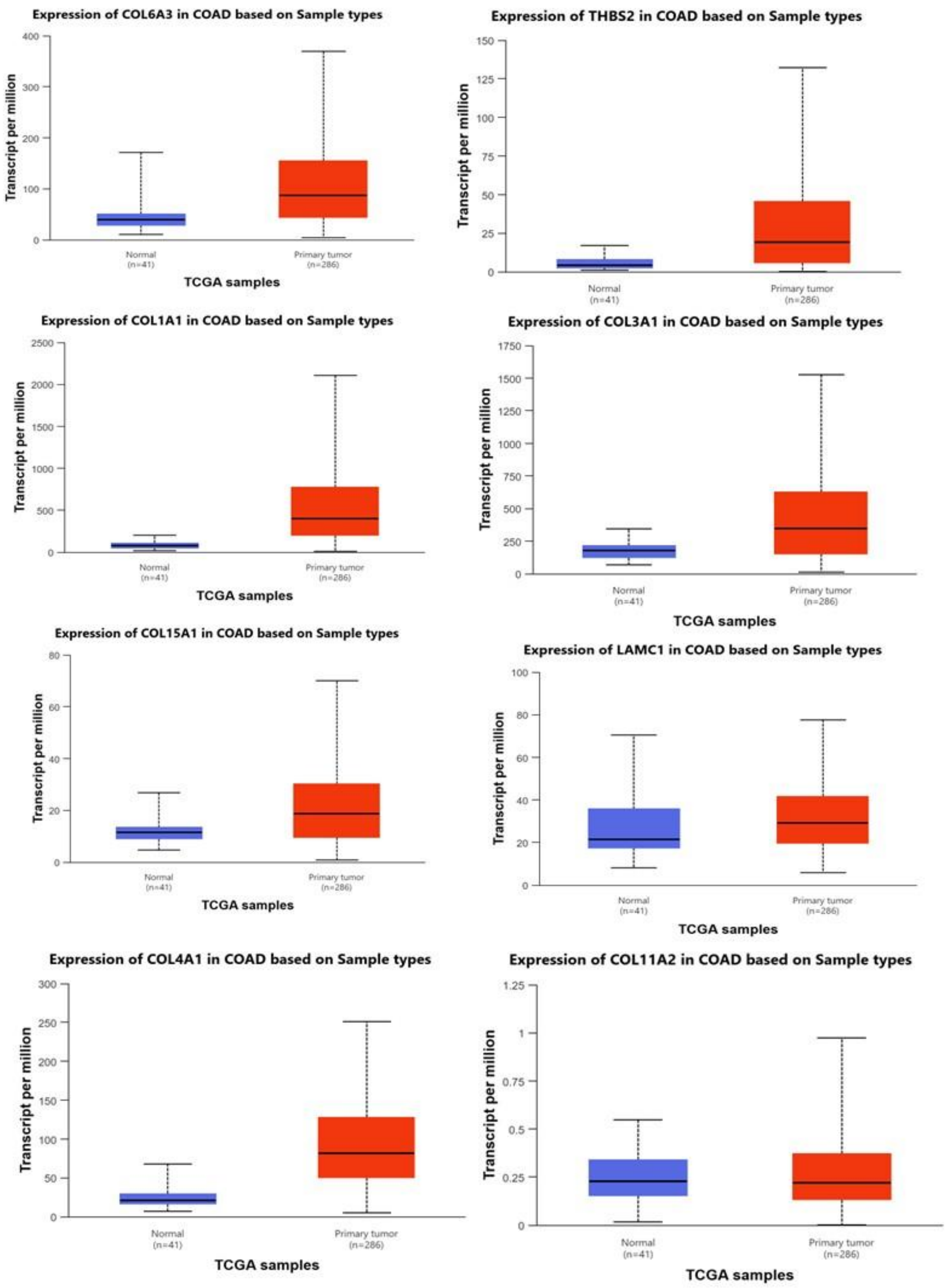

Fig. 1: Cross Validation. Differential gene expression of ML-ranked genes in normal versus colon tumour tissues by UALCAN webserver

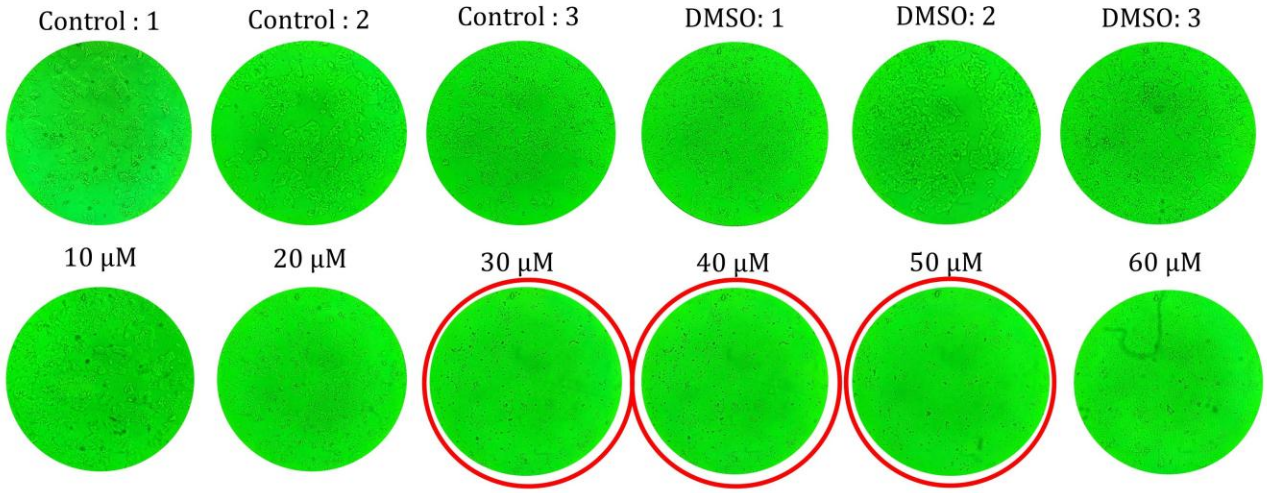

Fig. 2: Colon formation assay results with the concentration range of 10uM to 60uM Embelin on

Table. 1: Percentage of cell viability outcome from Colony formation assay

**Group**	**% Viability**
Control 1	96.60%
Control 2	99.60%
Control 3	103.80%
DMSO 1	98.30%
DMSO 2	103.10%
DMSO 3	101.60%
10 μM	97.40%
20 μM	68.60%
30 μM	64.90%
40 μM	58.20%
50 μM	53.40%
60 μM	67.50%
